# Supplementary material for: Modulation of sonochemical reactions by cavitation driven thermal degradation of aqueous salts solutions
Source: Commun Chem. 2026 Mar 5;9:160. doi: 10.1038/s42004-026-01961-4 (PMC13087145; doi:10.1038/s42004-026-01961-4)
Supplement: Supplementary file 2 — Supplementary informations [file 42004_2026_1961_MOESM2_ESM.pdf]

Supporting information of paper "Modulation of sonochemical reactions by cavitation driven thermal degradation of aqueous salts solutions"

A. Troia [1], M. Gallone[2], V. Vighetto[2], F. Pellegrino[3], S. Hernández [2], V. Cauda[2], V. Maurino[3]*

1 Ultrasounds & Chemistry Lab, Advanced Metrology for Quality of Life, Istituto Nazionale di Ricerca Metrologica (I.N.Ri.M.), Strada delle Cacce 91, Turin, 10135, Italy

2 Department of Applied Science and Technology, Politecnico di Torino, Corso Duca degli Abruzzi 24, Turin, 10129, Italy

3 Department of Chemistry, University of Torino, Via Giuria 7 10125, Torino, Italy

Supporting information

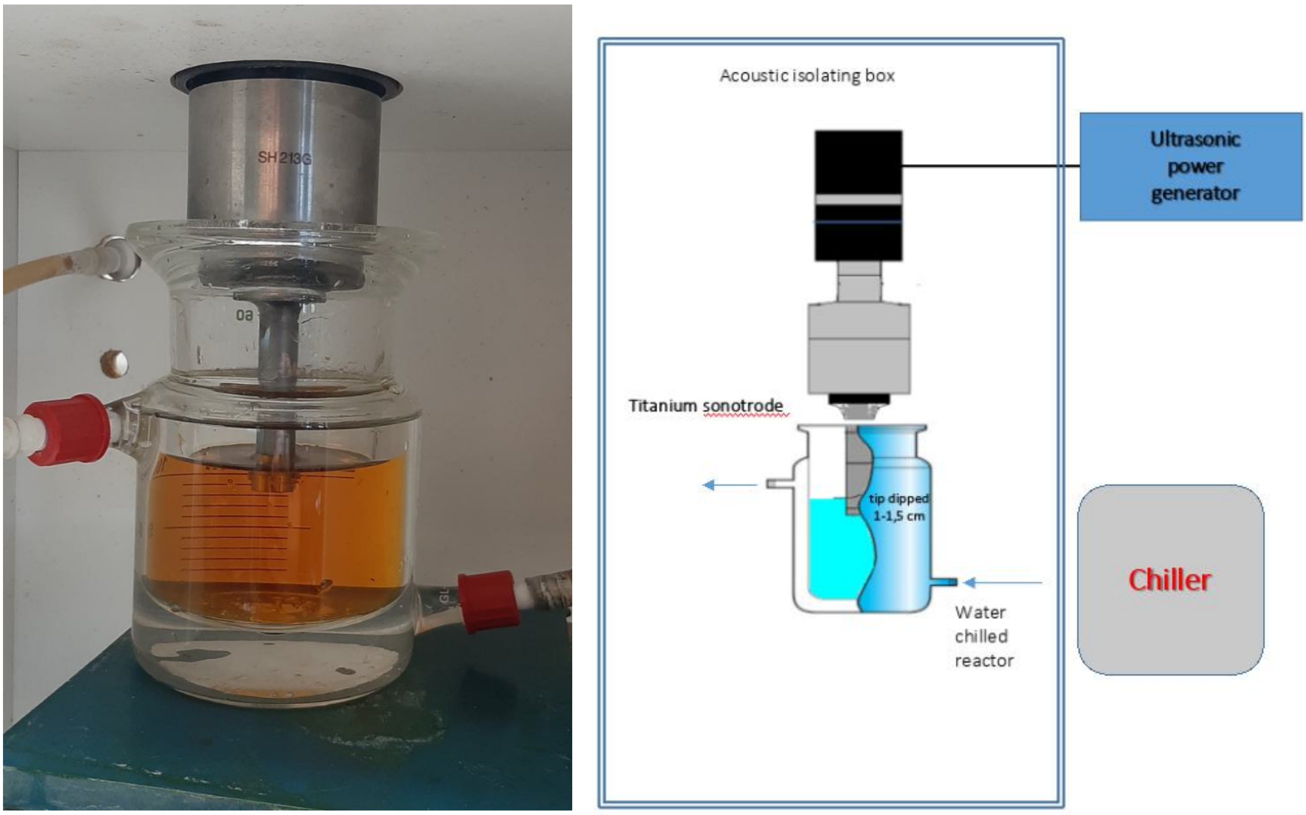

Figure S1 : *Picture of the 20 kHz sonochemical apparatus used for dye degradation experiments. 100 ml of aqueous dye solutions (MO in this picture) have been treated with Bandelin HD2000 Titanium sonotrode in water-cooled flask sonoreactors. The same set-up has been used for PL measurement with* Terephthalic *acid (TA). The temperature of sonicated solutions was maintained at 25°C. On the left a scheme of experimental apparatus*

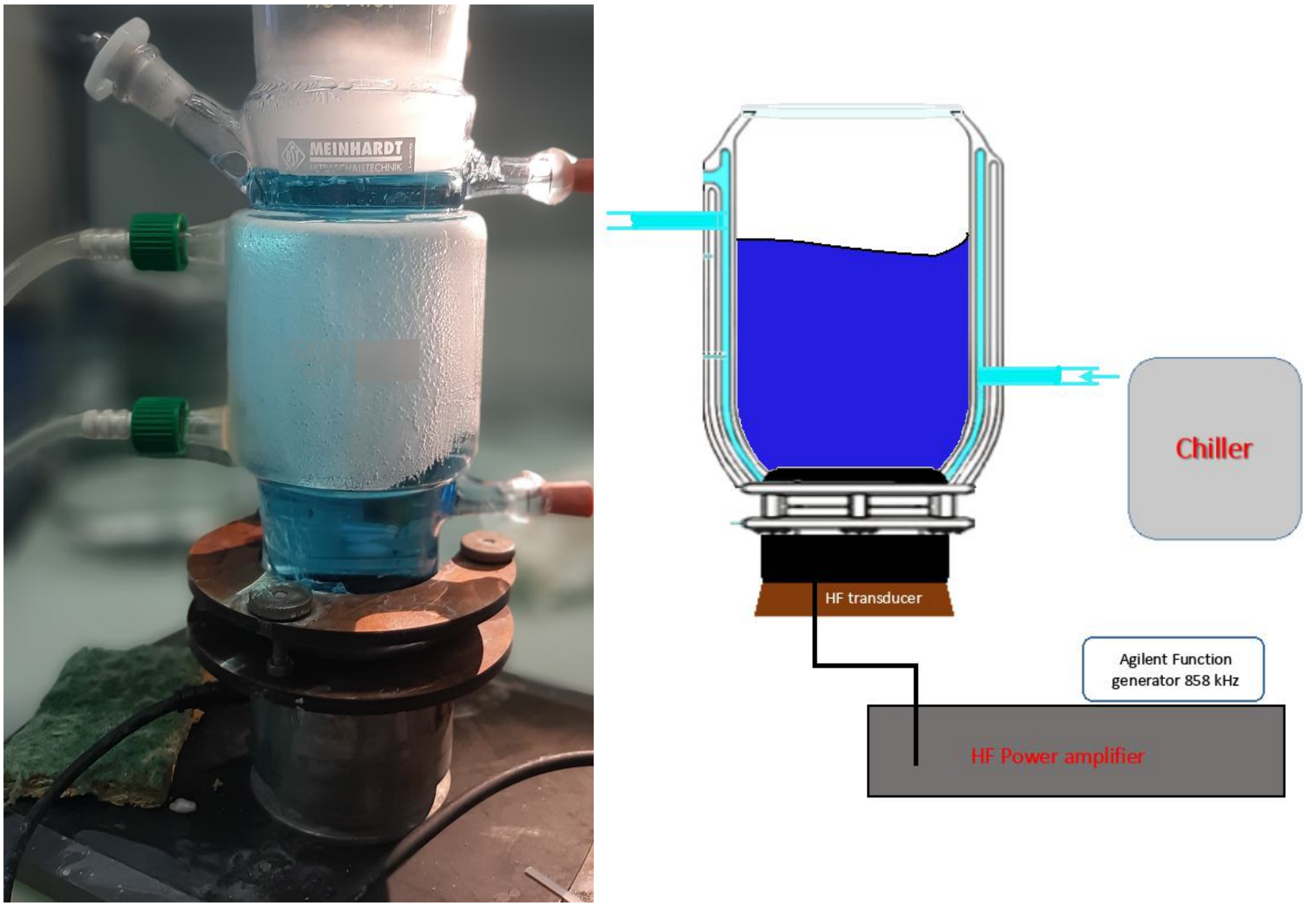

Figure S2: *Picture of the 858 kHz sonochemical apparatus used for dye degradation experiments. 250 ml of aqueous dye solutions (MB in this picture) have been treated with a Meinhardt multifrequency transducer using a chilling-cooled sonoreactor. The temperature of the sonicated solutions was maintained at 25°C. On the left a scheme of experimental apparatus.*

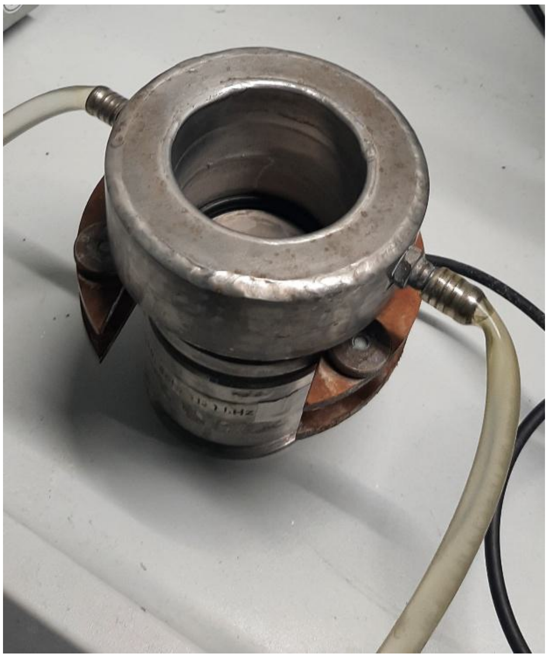

Figure S3 *Upper view of the high-frequency experimental setup for PL measurement. 100 ml of solution (TA conc. 30 ppm) in the presence of different salts was treated with a multifrequency transducer (Meinhardt) at 858 kHz in a stainless-steel cell equipped with a cooling jacket for 15 min. Acoustic pressure 15 bar (Muller- Platte hydrophone). The temperature of the sonicated solutions was maintained at 30°C.*

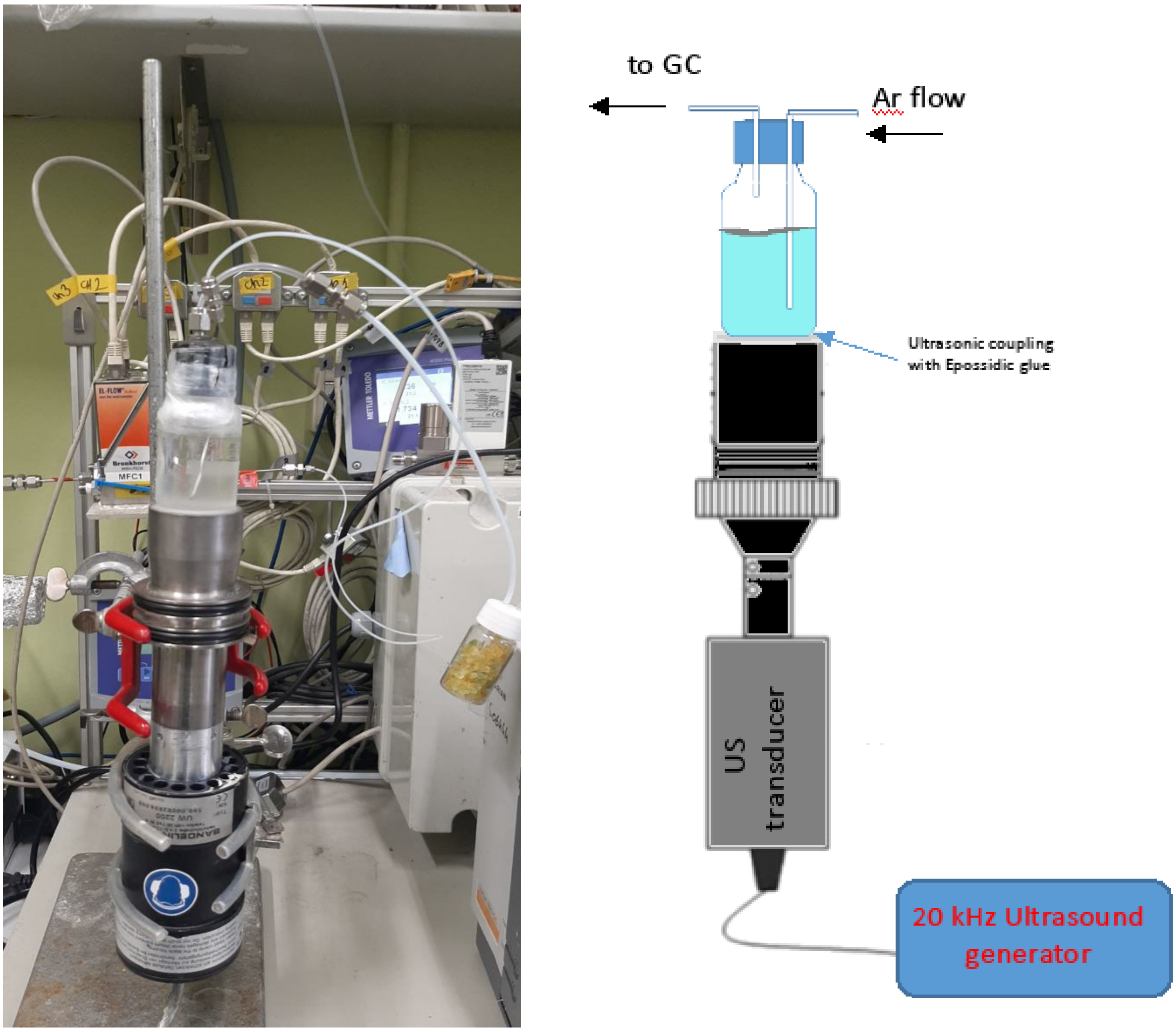

Fig S4: *Side view of the experimental setup for micro-GC measurement. 50 ml of 30% potassium sodium tartrate solution was treated with a 20 kHz ultrasound. The sealed vessel is glued to the bottom of Bandelin booster Sonoplus BB6 for indirect ultrasonic treatments, running continuously at 60% of maximum power, which corresponds to a power of. Argon* flow was used as a gas carrier. Gas samples were analysed every 3-5 minutes. *On the left a scheme of experimental apparatus.*

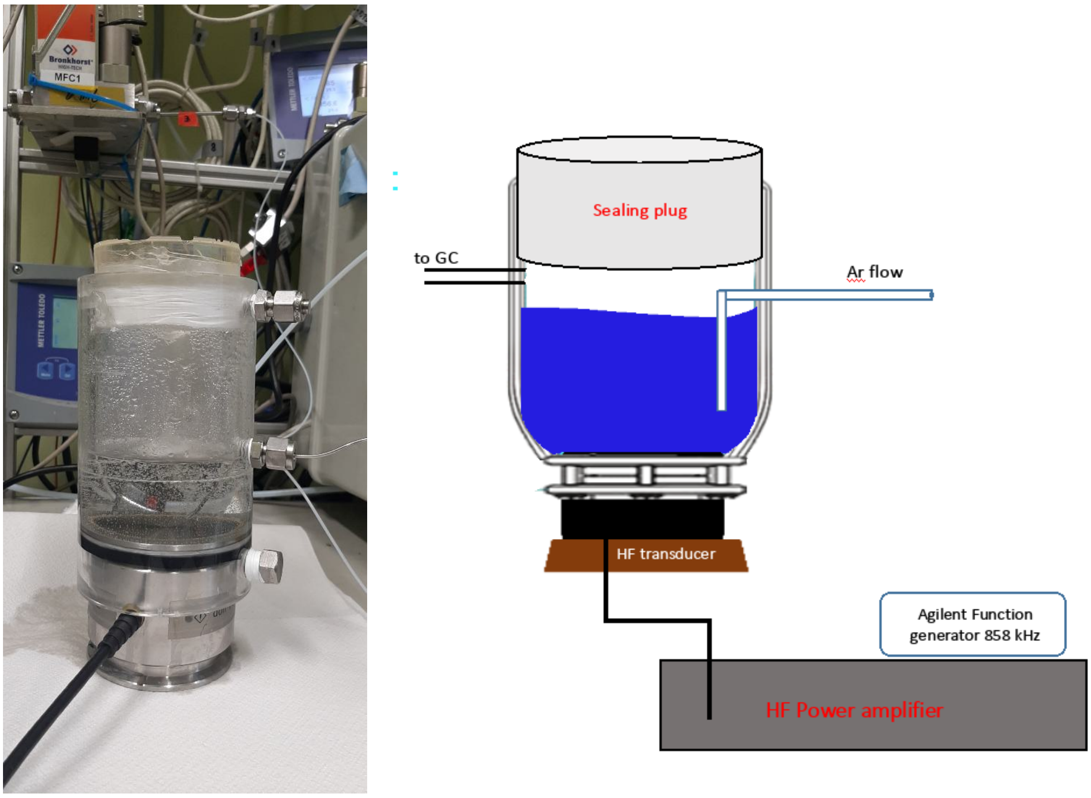

Figure S5 *Side view of the experimental setup for micro-GC measurement at high frequency. 200 ml of 30% Potassium sodium tartrate solution was treated at 858 kHz with an acoustic pressure of 13 bar. The sealed plastic vessel was sonicated from the bottom, with the ultrasonic transducer in direct contact with the solution. Gas samples were analysed every 3-5 minutes.* On the left a scheme of experimental apparatus.

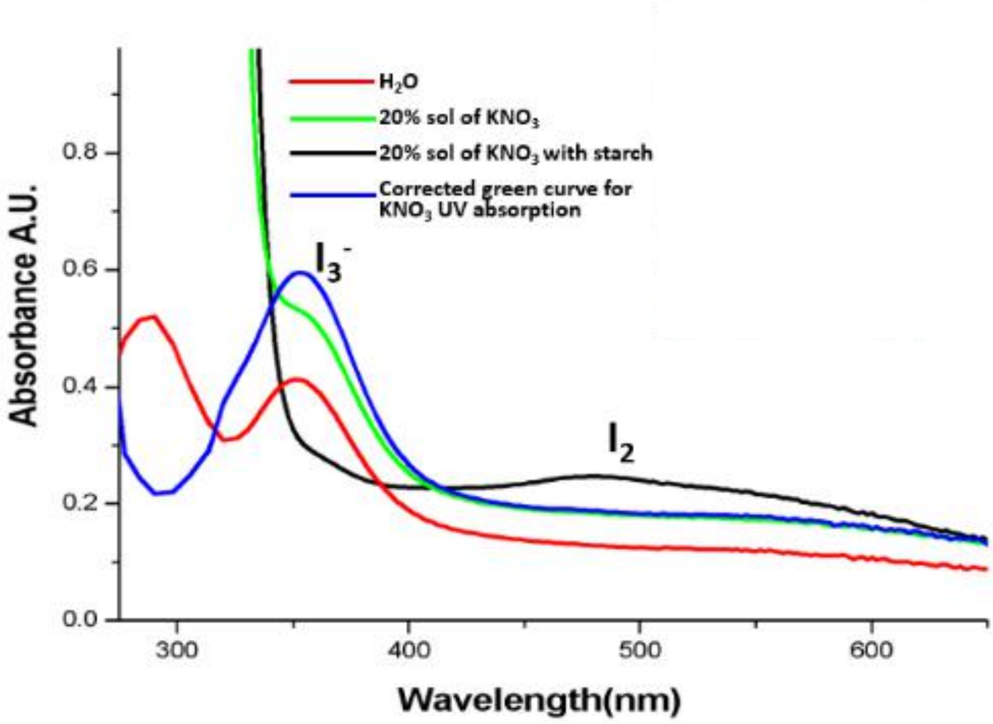

Figure S6 *UV-vis absorption spectra of KI 0.1 M solutions treated with 20 kHz for 15. Red line shows the increase of I_3^- in KI. Blue line is spectra for I_3^- of KI solution with KNO_3 (20%) solution; as a proper evaluation based on I_3^- absorption was affected by NO_3 UV vis absorption (green and black lines), we decided to carry out I_2 dosimetry, evaluating the absorbance of I_2-starch complex (visible in black line)*

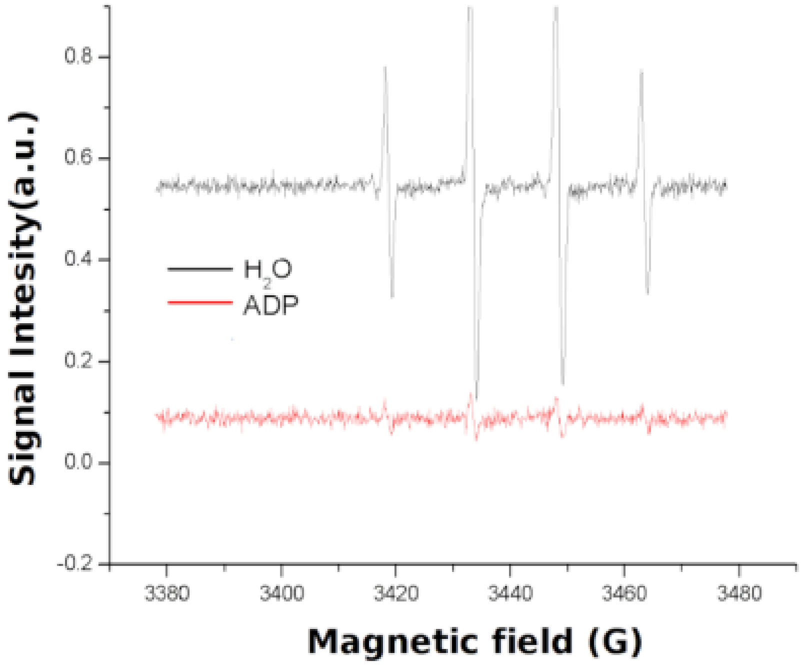

Figure S7 *EPR spectra of OH radical measured in water (black line) and ADP solution (conc. 5%) exposed for 5 min at 858 kHz.*

Reaction solution	Frequency [kHz]	Nominal power [W]	Gas	Reaction volume [L]	Catalysts	Concentration of catalysts [g/L]	Hydrogen production rate [µmol h^{-1} g^{-1}]	Reference
DI water	40	50	Not stated	0.15	TiO_2	0.5	17.3	Ref 28 of the study
DI water	40	50	Not stated	0.15	Au/TiO_2	0.5	288	Ref 28 of the study
DI water	40	100	Argon	0.01	Co,N nanowires	0.5	28.5	Ref 26 of the study
DI water	200	200	Argon	0.3	Co,N nanowires with N-defect TiO_2 rutile	0.7	250	Ref 27 of study
DI water	200	200	Argon	0.1	Commercial TiO_2 powder	2	103.1	Ref 27 of study
DI water	200	200	Argon	0.04	TiO_2 (Nippon Aerosil P-25)	5	210.6	Ref 27 of study
DI water	200	200	Argon	0.04	MnO_2	5	72.9	Ref 27 of study
DI water	780	5.13	Argon	0.0025	TiO_2 anatase	0.3	103.7	Current study, 2024
Seawater	40	100	Argon	0.01	Co,N nanowires	0.5	10.3	Ref 26 of the study
Seawater (4% NaCl solution)	200	200	Argon	0.3	Co,N nanowires with N-defect TiO_2 rutile	0.7	180	Ref 27 of study
Seawater	780	5.13	Argon	0.0025	TiO_2 anatase	0.3	54	Current study, 2024

Figure S8: Examples of Hydrogen generation using only ultrasound as an energy source and in the presence of catalysts as reported in recent paper [91]. *Our first experiments using Methylene Blue (MB) caused the reduction of MB to its leuco form which is colour less. Leaving the solution at air, after some hours its returned blue because of the oxygen diffusion
